# Evaluating Ortholog Prediction Algorithms in a Yeast Model Clade

**DOI:** 10.1371/journal.pone.0018755

**Published:** 2011-04-13

**Authors:** Leonidas Salichos, Antonis Rokas

**Affiliations:** Department of Biological Sciences, Vanderbilt University, Nashville, Tennessee, United States of America; Institut de Genetique et Microbiologie, France

## Abstract

**Background:**

Accurate identification of orthologs is crucial for evolutionary studies and for functional annotation. Several algorithms have been developed for ortholog delineation, but so far, manually curated genome-scale biological databases of orthologous genes for algorithm evaluation have been lacking. We evaluated four popular ortholog prediction algorithms (MultiParanoid; and OrthoMCL; RBH: Reciprocal Best Hit; RSD: Reciprocal Smallest Distance; the last two extended into clustering algorithms cRBH and cRSD, respectively, so that they can predict orthologs across multiple taxa) against a set of 2,723 groups of high-quality curated orthologs from 6 Saccharomycete yeasts in the Yeast Gene Order Browser.

**Results:**

Examination of sensitivity [TP/(TP+FN)], specificity [TN/(TN+FP)], and accuracy [(TP+TN)/(TP+TN+FP+FN)] across a broad parameter range showed that cRBH was the most accurate and specific algorithm, whereas OrthoMCL was the most sensitive. Evaluation of the algorithms across a varying number of species showed that cRBH had the highest accuracy and lowest false discovery rate [FP/(FP+TP)], followed by cRSD. Of the six species in our set, three descended from an ancestor that underwent whole genome duplication. Subsequent differential duplicate loss events in the three descendants resulted in distinct classes of gene loss patterns, including cases where the genes retained in the three descendants are paralogs, constituting ‘traps’ for ortholog prediction algorithms. We found that the false discovery rate of all algorithms dramatically increased in these traps.

**Conclusions:**

These results suggest that simple algorithms, like cRBH, may be better ortholog predictors than more complex ones (e.g., OrthoMCL and MultiParanoid) for evolutionary and functional genomics studies where the objective is the accurate inference of single-copy orthologs (e.g., molecular phylogenetics), but that all algorithms fail to accurately predict orthologs when paralogy is rampant.

## Introduction

Orthologous genes are homologs that originated by speciation events, whereas paralogs are homologs that originated by gene duplication events [1]. Accurate determination of orthologs and paralogs is fundamental to molecular evolution analyses, the first step in any comparative molecular biology study, and incredibly useful for functional prediction and annotation [2], [3], [4], [5], [6]. However, identifying orthologs and distinguishing them from paralogs is not always straightforward because genetic (e.g., gene duplications and losses) and population-level (e.g., hybridization and speciation) events can yield complex gene histories [2], [7].

The difficulty in accurately determining orthology, the utility of orthology in many different applications and disciplines, and the abundance of genomic data necessitating high-throughput pipelines for prediction, have led to the development of several different types of ortholog prediction algorithms [8]. For example, a number of graph-based algorithms use similarity searches, such as blast [9], to predict groups of orthologous genes (orthogroups), either in pairwise (between two taxa) or clustering (between multiple taxa) fashion [3], [6], [10], [11], [12], [13], [14], [15], [16], [17]. In contrast, tree-based algorithms predict orthogroups using explicit phylogenetic criteria [18], [19], [20], [21], [22], [23].

Although all these different types of ortholog prediction algorithms are widely used, studies that evaluate ortholog prediction algorithm performance for molecular phylogenetic purposes are not available. Furthermore, large-scale studies that evaluate the relative performance of a wide variety of different ortholog prediction algorithms have yielded contradictory results [10], [24], [25], [26]. For example, whereas Alexeyenko and co-workers [10] found that the graph-based MultiParanoid clustering algorithm produced the fewest errors, a different analysis showed that OrthoMCL, another graph-based clustering algorithm, had the best balance of sensitivity and specificity [27]. In contrast, Hulsen and co-workers [24] found that the InParanoid pairwise algorithm outperformed OrthoMCL in predictions of orthologous gene pairs. Furthermore, Altenhoff and Dessimoz [25] found that the graph-based OMA clustering algorithm [16] had the highest specificity (together with the homolog prediction algorithm HOMOLOGENE [28]), and that certain tree-based algorithms were occasionally outperformed by graph-based pairwise algorithms. Unfortunately, several differences in algorithm design make many of the above comparisons hard to interpret. For example, it is unclear how to interpret comparisons between pairwise and clustering ortholog prediction algorithms (e.g., [24]), or between algorithms that predict orthologs and paralogs (e.g., [25]), or how the results should be interpreted when the objective is not functional prediction but phylogenetic inference (e.g., [24]).

One potential explanation for these contradictory results might be that each one of the efforts to evaluate ortholog prediction algorithms makes assumptions likely to be violated [10], [24], [25], [27]. For example, several studies evaluated algorithms using functional similarity as a proxy for orthology [24], [25], whereas others evaluated algorithms against sets of orthologs identified by phylogenetic analysis [10], [25]. However, orthologous genes are not always functionally similar [2], and single-gene phylogenies frequently yield erroneous results [29], [30].

**Figure 1:**
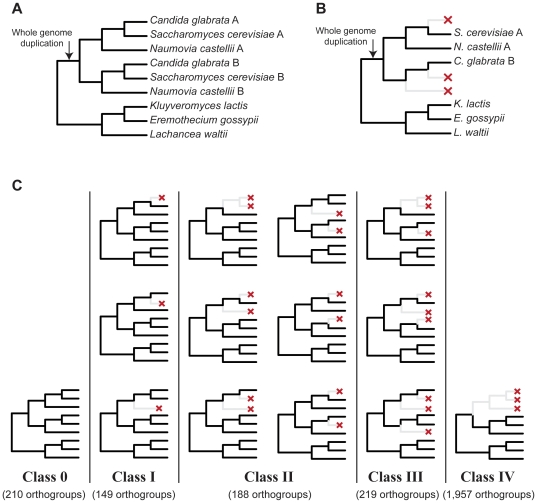
The generation of the five distinct classes of gene loss patterns following the yeast whole genome duplication (WGD). (**A**) Approximately 100 million years ago, the common ancestor of *S. cerevisiae, C. glabrata*, and *N. castellii* underwent WGD, resulting in the doubling of chromosomes. Segments that correspond to the two chromosome sets are known as tracks A and B. (**B**) An example of how the loss of paralogs from different tracks, if undetected, can generate an incorrect species tree. In the example, *C. glabrata* has lost a paralog from track A, whereas *S. cerevisiae* and *N. castellii* have lost paralogs from track B, ‘trapping’ ortholog prediction algorithms in incorrectly grouping the three post-WGD paralogs in an orthogroup. (**C**) In the aftermath of WGD, extensive loss of paralogs within homologous gene groups resulted in different gene loss patterns, known as classes 0 – IV [35]. Class 0 consists of groups that have not lost any paralogs. Groups in classes I and II have lost one and two paralogs, respectively. Finally, all groups in classes III and IV have lost three paralogs, however, all paralogs lost in class IV groups were on the same track (A or B).

The contradictory results in studies of ortholog prediction algorithm performance and the range of evaluation approaches developed suggest that there is a clear need for reliable reference genome-scale ortholog databases. One such high-quality reference database of homologous gene groups is the Yeast Gene Order Browser (YGOB) [31]. The YGOB is an excellent reference dataset for evaluating different ortholog prediction algorithms (e.g., [19], [32]) for two reasons. First, it contains genomes of varying evolutionary distances, and the homology of several thousand of their genes has been accurately annotated through sequence similarity, phylogeny, and synteny conservation data [31], [33]. Second, approximately 100 million years ago, a subset of species in the clade underwent a single round of whole genome duplication (WGD) (Figure 1A) [34]. Subsequent differential loss of gene duplicates originating from the WGD event resulted in groups of different gene retention pattern where in some cases the duplicates retained are paralogs [35] (Figure 1B), constituting ‘traps’ for ortholog prediction algorithms (e.g., Class III gene retention patterns in Figure 1C). Importantly, the YGOB database contains accurate ortholog annotations from species that predate and postdate the WGD event, as well as an accurate annotation of hundreds of such ‘trap groups’, allowing us to compare algorithm performance against orthogroup sets that are much more challenging to decipher.

Here, we evaluated the performance of four commonly used ortholog prediction algorithms – multiparanoid [10], orthomcl [3], rbh [4], [6], [12], [13], and rsd [14] in predicting orthogroups in six yeast proteomes by comparing their results against reference orthogroups retrieved from the YGOB database. To ensure that we evaluated all algorithms for their performance in detecting orthogroups across *multiple* species, we extended RBH and RSD into clustering algorithms (cRBH and cRSD, respectively). We selected these four algorithms among the several different ones available [8], based on their popularity, availability as standalone algorithms, and that they are not tree-based, which allows their implementation for downstream molecular phylogenetic analyses. We assessed the performance of each algorithm under a range of parameters and conditions, including in ‘traps’, as well as using varying numbers of species. We found that cRBH almost always outperformed all other algorithms, suggesting that simpler algorithms may often perform better than more complex ones in identifying orthologs across species, but that the false discovery rate of all algorithms was dramatically increased when groups of paralogs stemming from the WGD event were examined.

## Methods

### The Test Dataset

The test dataset consists of 31,012 proteins from the proteomes of the following six Saccharomycete yeasts: *Saccharomyces cerevisiae*, *Candida glabrata* (also known as *Nakaseomyces glabrata* [36]), *Naumovia castellii* (also known as *Saccharomyces castellii* [36]), *Lachancea waltii* (also known as *Kluyveromyces waltii* [36]), *Eremothecium gossypii* (also known as *Ashbya gossypii* [36]), and *Kluyveromcyes lactis* [37], [38], [39], [40], [41]. A common ancestor of three of these six yeast species (*S. cerevisiae*, *C. glabrata*, and *N. castellii*) underwent a single round of WGD (Figure 1A) [34]. Although the quality of annotations differs between the six species included in this study [31], it is unlikely to influence significantly our results. This is so because in our analyses we test all four algorithms on exactly the same data, and we have no reason to think that annotation quality differences would differentially affect the performance of ortholog prediction algorithms in our study.

### Constructing ‘Gold Groups’, a Reference Set of Orthogroups

The Yeast Genome Order Browser (YGOB) database is a manually curated homolog database of Saccharomycete proteins [31] from species that predate the WGD event (*K. lactis, L. waltii* and *E. gossypii*) as well as from species that postdate the WGD event (*S. cerevisiae, C. glabrata*, and *N. castellii*). Thus, for every chromosomal segment in the three pre-WGD species (*L. waltii*, *E. gossypii*, and *K. lactis*), assuming no loss, there are two corresponding chromosomal segments (known as track A and B) in the three post-WGD species. As a result, each homologous gene group in the YGOB database, assuming no gene loss, contains a single ortholog from each pre-WGD species, and two paralogs from each post-WGD species, one from track A and one from track B.

To construct a reference dataset of orthogroups deprived of paralogy we first retrieved all 2,723 annotated homologous gene groups from the YGOB (note that this set is a fraction of the total set of true orthogroups) and split each group into two subgroups. The first subgroup contained all ortholog genes from pre-WGD species together with all orthologs from post-WGD species found on track A, whereas the second subgroup contained the same orthologous genes from pre-WGD species together with all orthologs from post-WGD species found on track B. To avoid the double counting of orthologs from pre-WGD species in our assessment of ortholog predictions, we evaluated each prediction only against the subgroup that had the best match. We used these orthogroups, from here on referred to as ‘gold groups’, as the reference set to evaluate the performance of ortholog prediction algorithms.

### Ortholog Prediction Algorithms Tested

The MultiParanoid algorithm [10] is an extension of the graph-based InParanoid clustering algorithm [11], [42] for identifying orthologs and inparalogs across multiple species. InParanoid uses bi-directional best BLAST [9], [43] to identify putative orthologs and a clustering algorithm to identify their inparalogs. To do so, InParanoid assumes that any sequences from the same species that are more similar to the predicted ortholog than to any sequence from other species are inparalogs [11], [42]. MultiParanoid generates multi-species orthogroups by merging all pairwise InParanoid predictions, while minimizing the number of internal conflicts. Furthermore, the algorithm uses a ‘cut-off’ parameter based on the distance of candidate inparalogs to the predicted target ortholog to filter out weakly supported candidates. MultiParanoid was obtained from http://multiparanoid.sbc.su.se and InParanoid (version 3beta) was obtained upon request from inparanoid@sbc.su.se.

The OrthoMCL algorithm also builds upon the InParanoid algorithm [11], [42] by using the Markov Cluster (MCL) algorithm for predicting orthogroups across multiple species based on their sequence similarity information [3]. The algorithm uses an ‘inflation rate’ parameter, to regulate the ‘tightness’ of the predicted orthogroups. OrthoMCL (version 1.4) was obtained from http://orthomcl.org/common/downloads/software/v1.4/.

The Reciprocal Best Hit (RBH) algorithm [4], [6], [12], [13] relies on BLAST [9], [43] to identify pairwise orthologs between two species. According to the RBH algorithm, two proteins *X* and *Y* from species *x* and *y*, respectively, are considered orthologs if protein *X* is the best BLAST hit for protein *Y* and protein *Y* is the best BLAST hit for protein *X*. We integrated a ‘filtering’ parameter *r* that enabled us to avoid constructing orthogroups that contained distant homologs by considering the degree by which the two proteins differed in sequence length or BLAST alignment [44], [45]. Thus, putative orthogroups are retained if:
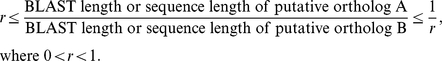



From the above equation, it follows that *r* values close to 1 are likely to filter out a larger number of putative orthologs, whereas *r* values close to 0 are likely to include all putative orthologs. The default mode of the algorithm does not use the filtering parameter *r*.

The Reciprocal Smallest Distance (RSD) algorithm [14] generates global sequence alignments for a small number of top BLAST hits against a query gene *X* from species *x*. RSD then calculates the maximum likelihood evolutionary distance between *X* and its top BLAST hits, identifying the gene with the smallest evolutionary distance from *X* (e.g., gene *Y* from species *y*). If the RSD search using gene *Y* from species *y* as the query also identifies gene *X* from species *x* as its closest relative, then proteins *X* and *Y* are considered orthologs [14], [15]. In RSD, the user can modify the shape parameter *a* of the gamma distribution, a key determinant of the estimated evolutionary distance between genes. The RSD algorithm was obtained from http://roundup.hms.harvard.edu/site/.

### Extending the Pairwise RBH and RSD Algorithms into Clustering Algorithms cRBH and cRSD

To directly compare the clustering performance of all four ortholog prediction algorithms we extended the pairwise algorithms RBH and RSD into clustering algorithms cRBH and cRSD, respectively. cRBH and cRSD construct orthogroups from more than two species as follows (see also [46]). Considering all pairwise BLAST similarity searches for genes *A*, *B, C,…, N-1, N* from species *a*, *b, c,…, n-1, n* to form an orthologous gene group, gene *B* must be the reciprocal best hit to gene *A*, gene *C* the reciprocal best hit to gene *B* or gene *A*, …, and gene *N* the reciprocal best hit to any gene 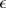
[*A, B, C,…, N-1*]. In cases such as when gene *A* from species *a* is the reciprocal best hit to gene *B* from species *b* and to gene *C_1_* from species *c*, but gene *B* is the reciprocal best hit to gene *C_2_* from species *c*, the algorithm drops species *c* from the orthogroup.

### Evaluating the Performance of Ortholog Predictions

We used a BLASTP cut-off *E*-value of ≤ 1e^-5^ in all orthogroup predictions made with all four algorithms. We run the MultiParanoid algorithm using a range of cut-off parameter values (cut-off  =  {0.0, 0.01, 0.05, 0.1, 0.2, 0.3, 0.4, 0.5, 0.6, 0.7, 0.8, 0.9}; 0.0 is the default value), the OrthoMCL algorithm using a range of inflation rate parameter values (inflation rate =  {0.1, 0.5, 1.0, 1.5, 2.0, 2.5, 3.0, 3.5, 5, 7.5, 10.0, 100.0}; 1.5 is the default value), the cRBH algorithm by ranging the values assigned to the filtering parameter *r* (*r* =  {no *r*, 0.1, 0.2, 0.3, 0.4, 0.5, 0.6, 0.7, 0.8, 0.9}; no *r* is the default option), and the cRSD algorithm by ranging the values of the shape parameter *a* (*a* =  {0.1, 0.4, 0.5, 0.6, 0.7, 1.0, 1.5, 2.0, 2.5, 5.0}; 0.5 is the default value). For each algorithm and its range of parameter values, we calculated its accuracy, sensitivity, specificity, and false discovery rate using the following equations:
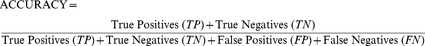


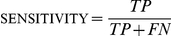


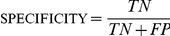


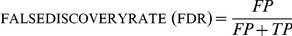



Finally, we graphically plotted the receiver operating characteristic (roc curve) of sensitivity versus (1 − specificity).

### The Evaluation Pipeline for Test Orthologous Genes and Orthogroups

**Figure 2:**
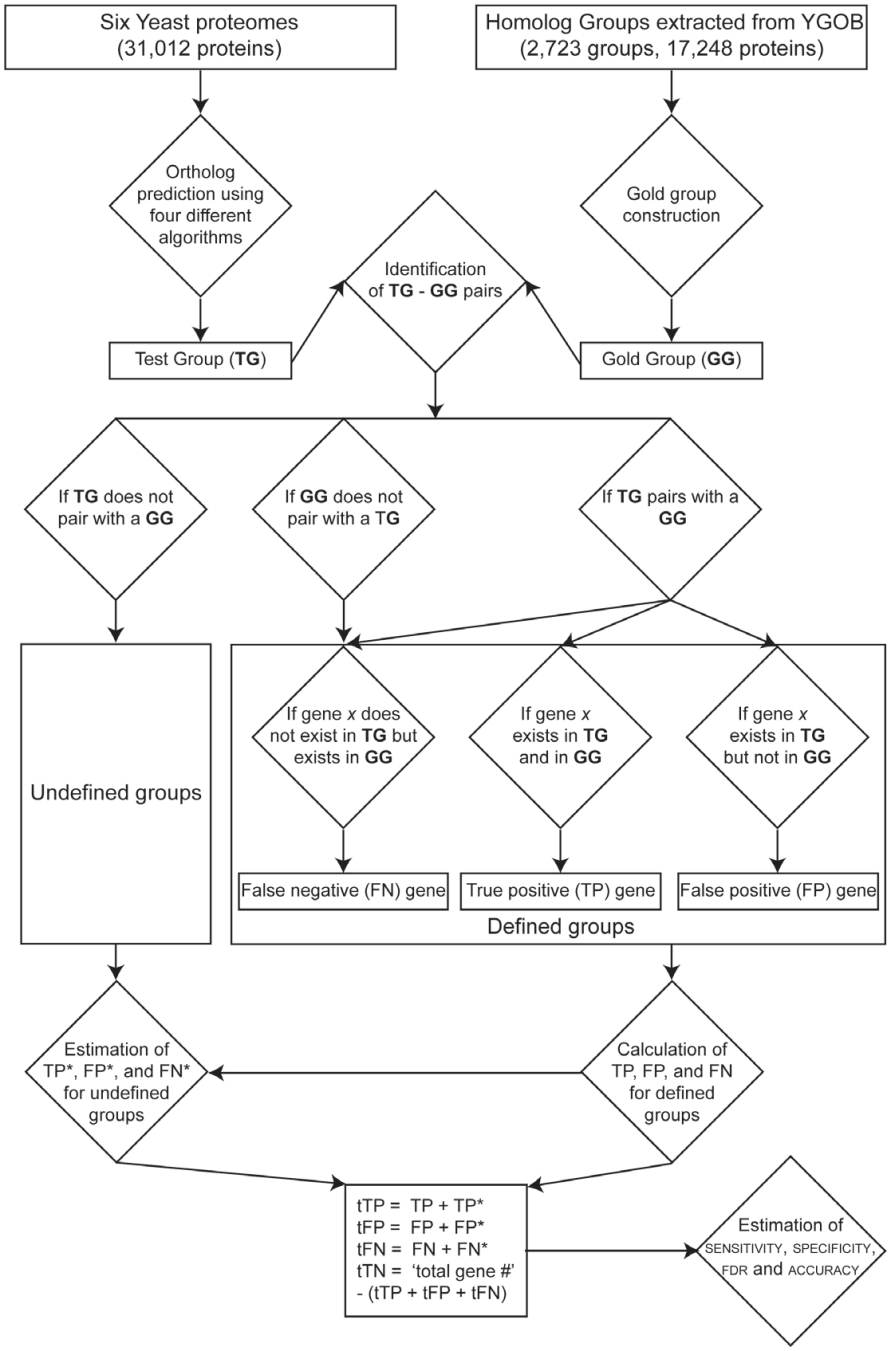
The pipeline used to evaluate the performance of the ortholog prediction algorithms. The pipeline evaluates algorithm performance by comparing their predictions on six yeast proteomes against a high-quality reference set of orthologs (gold groups) constructed from the YGOB [31]. The pipeline first compares each test group against the set of gold groups. If the test group matches with a corresponding gold group, the test group is characterized as ‘defined’ and the two groups are further compared on a gene-by-gene basis. If there is no match, the test group is characterized as ‘undefined’. For the ‘defined’ groups, genes present in both the test and the gold groups are considered true positives (TP), whereas genes present only in the test group or only in the gold group are considered as false positive (FP) and false negative (FN), respectively. From the TP, FP, and FN values for all ‘defined’ groups we then estimated the true positives (TP*), false positives (FP*), and false negatives (FN*) for the ‘undefined’ set of groups. Finally, by adding the values obtained from the analysis of ‘defined’ and ‘undefined’ groups we calculated the total number of true positive (tTP), false positive (tFP), false negative (tFN), and true negative (tTN) genes for all test groups, and used them to estimate each algorithm's sensitivity, specificity, accuracy and false discovery rate (See Methods and Text S1).

We evaluated the ability of each ortholog algorithm to predict orthogroups by comparing their predictions against the reference gold groups. According to our evaluation pipeline (Figure 2 and Text S1), each predicted orthogroup was first compared against the set of gold groups to identify, if any, its corresponding gold group. If a test group shared at least two genes with a reference gold group, the test group was characterized as a ‘defined’ test group. In all other cases, the test group was considered ‘undefined’.

For the defined orthogroups, we considered all genes shared between the test group and its corresponding gold group as true positive (TP), and any genes in the test group that did not also belong to the gold group as false positive (FP) (Figure 2 and Text S1). FP genes could belong to a different gold group or to be absent from the set of corresponding gold groups. Finally, we considered all those genes present in gold groups that did not belong to any test groups as false negative (FN).

Given that the number of reference gold groups is much smaller than the total number of true orthogroups in our dataset, we expect that a significant number of test orthogroups will not have corresponding gold groups, and hence will be undefined. Because we wanted to calculate values that were representative for the entire dataset, we estimated the number of true positive (TP*), false positive (FP*), and false negative (FN*) for the undefined orthogroups by multiplying the number of TP, FP, and FN calculated from the defined groups with the ratio of the number of undefined genes on the number of defined genes (Figure 2 and Text S1). For example, TP* is the product of the TP value multiplied by the ratio of the number of undefined genes on the number of defined genes. Finally, by calculating the total number of true positive (tTP  =  TP + TP*), false positive (tFP  =  FP + FP*), and false negative (tFN  =  FN + FN*) genes, we were able to estimate the number of total true negative genes (tTN =  total number of genes – tTP – tFP – tFN) in our dataset (Figure 2 and Text S1).

To ensure that the calculated TP, FP, and FN values for proteins that belonged to ‘defined’ groups were also representative of the remainder of the proteins (i.e., those that belong to the ‘undefined’ groups) (Figure 2), we tested whether *S. cerevisiae* genes that belong to ‘defined’ and ‘undefined’ groups differed significantly in evolutionary rate (measured by the *d_N_/d_S_* ratio), number of paralogs in genome, and codon adaptation index. We obtained the data for evolutionary rate and codon adaptation index calculations from the study of Wall *et al.* [47]. We calculated the number of *S. cerevisiae* paralogs per protein using BLASTP [9]. To evaluate whether the evolutionary and functional properties of genes that belong to the ‘defined’ and ‘undefined’ groups were statistically significant, we performed a two-tailed t-test (assuming unequal variance and unequal sample size) [48].

### Evaluating Algorithm Performance for Varying Numbers of Species

To evaluate the performance of each algorithm across varying numbers of species, we examined all possible combinations for three, four, and five yeast proteomes and calculated each algorithm's accuracy and fdr. All algorithms were run using the parameter values that yielded the highest accuracy in orthogroup prediction on the six yeast proteomes dataset.

### Evaluating Algorithm Performance against Different Classes of Gene Loss Events

Our reference dataset contains orthogroup classes where some of the homologs retained are paralogs. To investigate how each algorithm performed in these ‘trap groups’, we divided the 2,723 gold groups into the five classes described by Scannell *et al.* [35] (Figure 1C) and calculated the accuracy and fdr for each algorithm. All algorithms were run using the parameter values that yielded the highest accuracy in orthogroup prediction on the six yeast proteomes dataset.

## Results

**Figure 3:**
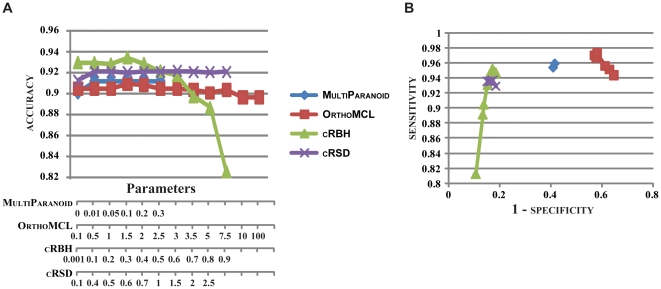
The accuracy and receiver operating characteristic (roc) curve for each ortholog prediction algorithm across a range of parameter values. (**A**) The accuracy [(TP + TN)/(TP + TN + FP + FN)] of each ortholog prediction algorithm (shown on the Y-axis) is plotted against the range of algorithm-specific parameter values (shown on the X-axis). Values for MultiParanoid are for the ‘cut-off’ parameter, values for OrthoMCL are for the ‘inflation rate’ parameter, values for cRBH are for the ‘filtering parameter *r*’, and values for cRSD are for the ‘shape parameter *a*’. (**B**) The roc curve for each ortholog prediction algorithm shows sensitivity [TP/(TP + FN)] (on the Y-axis) plotted against 1 – specificity [1 – (TN/(TN + FP))] (on the X-axis). Optimal values and distributions reside on the top left of the graph. All values depicted in the graphs are shown in Table S1.

**Figure 4:**
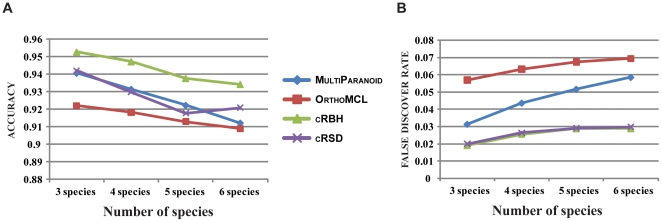
The accuracy and fdr of ortholog prediction algorithms using varying numbers of species. (**A**) The accuracy of ortholog prediction algorithms (shown on the Y-axis) is plotted against varying numbers of species (shown on the X-axis). (**B**) The fdr of ortholog prediction algorithms (shown on the Y-axis) is plotted against varying numbers of species (shown on the X-axis). Each algorithm was run using the parameter value yielding the highest accuracy. All values depicted in the graphs are shown in Table S1.

**Figure 5:**
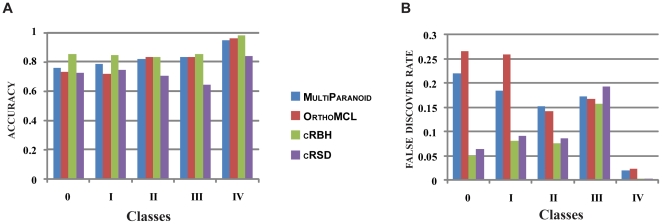
The accuracy and fdr of ortholog prediction algorithms across five orthogroup classes with different gene retention patterns. The five classes are described in Figure 1. (**A**) The accuracy of ortholog prediction algorithms (shown on the Y-axis) is plotted against the five classes (shown on the X-axis). (**B**) The fdr of ortholog prediction algorithms (shown on the Y-axis) is plotted against the five classes (shown on the X-axis). Each algorithm was run using the parameter value yielding the highest accuracy. All values depicted in the graphs are shown in Table S1.

**Figure 6:**
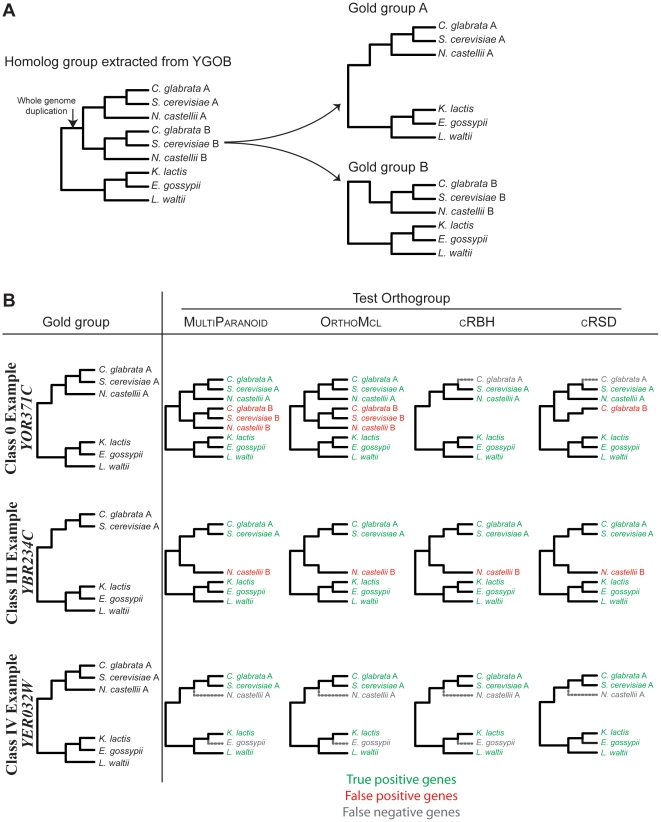
Examples of the behavior of the four algorithms in predicting orthogroups from gold groups belonging to three different classes. (**A**) Construction of gold groups (gold groups A and B) from the set of homologous gene groups from the YGOB. Each test group is evaluated against only against the gold group that had the best match. (**B**) The orthogroups for three different gold groups belonging to classes 0, III and IV predicted by the four different algorithms. The gold group is shown on the left-most column. The *S. cerevisiae* gene name for each of the three gold groups is shown on the left. Genes correctly predicted as belonging to each orthogroup (true positives) are shown in green, genes incorrectly predicted as belonging to each orthogroup (false positives) are shown in red, whereas genes present in a gold group that were not predicted to belong to this or any other test group (false negatives) are shown in grey.

We evaluated the performance of four different algorithms (MultiParanoid, OrthoMCL, cRBH and cRSD) in predicting orthogroups against a manually curated, high-quality database of ortholog groups (gold groups), by estimating sensitivity, specificity, accuracy and fdr across different parameter values, using a varying number of species and across different gene loss classes (Figures 3, 4, 5, 6 and Table S1). *S. cerevisiae* genes that belong to ‘defined’ and ‘undefined’ groups did not differ significantly in evolutionary rate, number of paralogs in genome, and codon adaptation index (all *p*-values for all measures across all algorithms were larger than 0.05). Thus, the ‘defined’ and ‘undefined’ orthogroups do not differ significantly. Therefore, our estimation of the number of true positive (TP*), false positive (FP*), and false negative (FN*) for the undefined orthogroups based on the number of TP, FP, and FN calculated from the defined groups seems to be valid and our results should be representative of the entire population of orthogroups present in the six yeast genomes under study.

### Comparing Algorithm Performance across Different Parameter Values

Ranging the cut-off parameter value of the MultiParanoid algorithm had minor effects on its performance. All analyses with cut-off values >0 yielded identical results with higher sensitivity and accuracy, but lower specificity relative to the default cut-off value of zero. The OrthoMCL algorithm did not exhibit any clear trade-off between sensitivity and specificity with increasing inflation rate values. Specifically, predictions using inflation rate values ≥3.5 had both lower sensitivity and specificity. The algorithm had almost equal sensitivity for values <3, with the best specificity and accuracy obtained when the inflation rate was 1.5. The cRBH algorithm had the highest sensitivity and accuracy when *r* was 0.3, although similar values were obtained when *r* was not set (default) or when *r* was 0.4. In general, *r* values greater than 0.4 decreased the sensitivity of the algorithm by excluding increasing numbers of putative orthologs, but increased its specificity. For cRSD, sensitivity and accuracy remain largely stable and optimal for *a* values ≥0.4. sensitivity was highest at *a* = 0.4, whereas accuracy and specificity were both highest at *a* = 1.5. In general, the algorithm produced a limited number of false positives, which resulted in both high accuracy and low fdr.

The performance of all ortholog algorithms across different parameter values is summarized in Figure 3. Our results suggest that cRBH is the most accurate algorithm. Specifically, cRBH had the highest accuracy (0.934, for *r* = 0.3), followed by cRSD (0.921, for *a* = 1.5), MultiParanoid (0.912, for any cut-off >0) and OrthoMCL (0.909, for inflation rate  = 1.5) (Figure 3). Higher sensitivity is typically associated with either higher numbers of true positives or lower number of false negatives. Across the range of all parameters for all algorithms, OrthoMCL showed the highest sensitivity (inflation rate  = 1), followed by cRBH (*r* = 0.3), multiparanoid (for cut-off >0) and cRSD (for *a* = 0.4) (Figure 3). In contrast, higher specificity is typically associated with lower numbers of false positives. Across the range of all parameters for all algorithms, cRBH has the highest specificity (for *r* = 0.9), followed by cRSD (for *a* = 0.1), MultiParanoid (for cut-off = 0) and OrthoMCL (for inflation rate  = 1.5) (Figure 3).

### Comparing Algorithm Performance Using a Varying Number of Species and across Different Gene Loss Classes

To evaluate the performance of each algorithm under a varying number of species, we ran the algorithms for all possible combinations of three, four and five species (Figure 4). Once again, cRBH had the highest accuracy (Figure 4A) and the lowest fdr across all taxon numbers (Figure 4B), followed by cRSD.

To investigate how the existence of ‘trap’ gold groups affected the performance of the four ortholog prediction algorithms, we compared their accuracy and fdr across the five different gold group classes (Figure 1C). Overall, all four algorithms had higher fdr values in paralog-containing classes (classes 0 through III) than in paralog-lacking classes (class IV) (Figure 5). cRBH had the highest accuracy and the lowest fdr values across all classes. However, not all algorithms exhibited the same behavior across the five classes. For example, whereas cRBH and cRSD had their highest fdr values in class III, OrthoMCL and MultiParanoid had their highest fdr values in class 0, due to the larger number of paralogs (Figures 5, 6). Finally, note that in class IV, where all paralogs from the same track (track A or B) have been lost, all algorithms perform well, but cRBH still showed the highest accuracy and the lowest fdr.

## Discussion

More than twenty orthology prediction algorithms and databases have been developed, which can be divided into three main groups: graph-based (orthology is inferred from sequence similarity), tree-based (orthology is inferred from phylogeny), and hybrid-based (orthology is inferred from both phylogeny and sequence similarity) [8]. In this study, we compared the performance of four popular graph-based clustering algorithms (MultiParanoid, OrthoMCL, cRBH and cRSD) that predict orthogroups for use in molecular phylogenetics. We did not include tree-based and hybrid algorithms because ortholog prediction on large datasets typically requires faster algorithms, and because the reliance of these algorithms on knowledge of the gene family (e.g., [18]) or species phylogeny (e.g., [19]) can render them inappropriate for downstream phylogenetic studies (but see [49]). Furthermore, the use of YGOB as our reference dataset required the availability of standalone algorithms that could make predictions on user-provided datasets.

For the majority of orthogroup predictions, all methods showed high accuracy and low fdr (Figures 3, 4, 5), a finding consistent with their similarity in algorithm construction and popularity in the literature. However, our results also suggested that cRBH outperformed all other three algorithms in almost all of our comparisons (Figures 3, 4, 5). These results directly pertain to on-going debates about the choice of ortholog prediction algorithms for downstream evolutionary, genomic and functional analyses [8], [10], [24], [25], [26]. However, the selection of the optimal ortholog prediction algorithm for inferring orthologous genes and groups across such a remarkably wide range of fields and applications is a complex problem that is likely to be influenced by many parameters.

### Curated Ortholog Databases as Gold Standards for Algorithm Evaluation

Several different benchmarks have been used to assess the accuracy of ortholog prediction algorithms [8]. However, the lack of ‘gold’ standard reference datasets has made interpretations of relative performance challenging. For example, several recent comparative studies have yielded contradictory results [10], [24], [25], [26], but the degree to which this lack of common high-quality reference sets contributes to these conflicts is largely unknown. To circumvent these issues, we employed a highly accurate genomic database of homologs to evaluate directly ortholog prediction algorithms (see also [19], [32]). We think that our gold group set has strong potential to become one such ‘gold’ standard for the evaluation of ortholog prediction algorithms. Of course, our dataset stems from species inhabiting a single small twig of the tree of life. Thus, it remains an open question whether these results hold across branches of the tree of life, or whether accuracy in ortholog prediction in different branches will require several different approaches. As more genomes from several clades of the tree of life are sequenced [50] we anticipate that highly accurate homolog databases, like the YGOB [31], will become commonplace and more densely populated with orthologs from several additional species (e.g., [51]), thus greatly facilitating algorithm evaluation and testing the generality (or not) of findings such as those reported in this study.

One potential limitation of such reference databases is that their construction might be possible only from genomes of close relatives. This is so, because accurate annotation of orthologs between distantly related species is much more challenging; at greater evolutionary distances protein homology is frequently reduced to homology between domains [52], domain shuffling is commonplace [53], and independent data, such as synteny conservation, that are highly informative for accurate annotation of orthologs between closely related species, become less useful [54]. Nevertheless, our findings (see also [19], [32]) suggest that evaluation approaches against high-quality ‘gold standard’ databases [31], [51] are likely to be a very useful addition to existing benchmarks [8], [24], [25] in the quest to accurately infer orthologs on a genome-wide scale.

### Simpler Algorithms Can Sometimes Be Better

The usefulness of ortholog identification in several downstream genomic, molecular and evolutionary analyses, coupled with the abundance of genomic data from diverse organisms, has spurred the development of several ortholog prediction algorithms [8]. Thus, we were surprised to find that cRBH, a conservative clustering version of the simplest and earliest-developed of the four algorithms tested that drops instead of resolving inconsistencies [4], [6], [12], [13], [55], was consistently (e.g., across several parameter values and varying numbers of species) the best ortholog predictor. In agreement with our results, a recent phylogenetic and functional assessment of ortholog prediction algorithms and databases also found that RBH performed well and its predictions were, in several instances, better than those of more complex algorithms [25].

The superior performance of cRBH and cRSD may be partially explained by the fact that OrthoMCL and MultiParanoid are designed to also include inparalogs in their orthogroup predictions (Figure 6). Using our evaluation pipeline, this design can raise significantly the number of false positives, thus decreasing the algorithms' accuracy and specificity, but increasing the algorithms' FDR and sensitivity. However, when the algorithms were tested on class IV orthogroups, which comprise the majority of gold groups (1,957 orthogroups or ∼70%) and have lost all paralogs from the same track (Figure 1C), cRBH still performed better by showing a very low fdr, high accuracy and specificity and almost equal sensitivity as OrthoMCL, the most sensitive algorithm (Figure 3). Although this difference in performance could be due to the inclusion of other paralogs that did not originate through the WGD, the existence of other paralogs is unlikely to account fully for it. For example, analysis of a dataset that contained only genes belonging to class IV gold groups, an inparalogs-free dataset, also showed that cRBH and cRSD have the highest accuracy and lowest fdr. Finally, the set of single-copy orthogroups obtained from OrthoMCL and MultiParanoid is much smaller than the total number of predicted orthogroups and shows much lower sensitivity and accuracy. This suggests that the popular approach of using these algorithms for orthogroup prediction in molecular phylogenetic studies is less accurate than the use of algorithms designed to predict orthogroups that contain a single gene from each species, like cRSD and cRBH.

When tested on the class III groups (Figure 1), in which the pattern of gene loss forced all algorithms to place single-copy paralogs in the same orthogroup, all algorithms showed very high fdr values (Figures 1, 5). cRBH was again the best performing algorithm, partly due to the effect of the filtering parameter *r* in dropping putative orthogroups composed of distantly related paralogs. Note that the lack of a ‘gold’ reference dataset or the adoption of an evaluation strategy based on majority-rule predictions would have not permitted us to identify the failing of these algorithms for class III orthogroups, and would have instead considered most of them as likely true.

### Choosing the Right Algorithm for Orthologous Gene Group Prediction

Our results suggest that simpler algorithms, like cRBH and cRSD, might be better choices for many downstream evolutionary analyses than more complex ones in cases where the objective is to identify orthogroups and that the trend of several studies toward using more complex ortholog prediction strategies is not always justified. One of the criteria used in our selection of algorithms was for ones whose orthogroup predictions would be appropriate for use in phylogenetic analyses. Thus, we did not evaluate tree-based or hybrid-based algorithms. However, such algorithms could be much more appropriate for orthogroup prediction in several other contexts, e.g., for functional annotation. For example, the SYNERGY algorithm [19], [56], which integrates information from similarity searches, gene trees, and synteny in its orthogroup predictions has been shown to be more accurate than RBH [19], and likely to be a much better choice for evolutionary genomics and functional studies. Similarly, because RBH, RSD and their clustering extensions are limited to finding orthogroups that contain a single gene from each species, they will fail to detect the presence of inparalogs, and in contrast to algorithms such as SYNERGY [19], [56], MultiParanoid [10] and OrthoMCL [3], are probably of no use for studying gene family evolution.

## Supporting Information

Table S1 The accuracy, sensitivity, specificity and fdr values of ortholog prediction algorithms across a range of parameter values (S1A), using varying numbers of species (S1B), and across five orthogroup classes with different gene retention patterns (S1C).
(XLS)

Text S1 Analytical description of the evaluation algorithm.
(DOC)
