# Supplementary material for: Evaluating Ortholog Prediction Algorithms in a Yeast Model Clade
Source: PLoS One. 2011 Apr 13;6(4):e18755. doi: 10.1371/journal.pone.0018755 (PMC3076445; doi:10.1371/journal.pone.0018755)
Supplement: Text S1 — Analytical description of the evaluation algorithm. (DOC) [file pone.0018755.s002.doc]

**Text S1.Analytical description of the evaluation algorithm.** For the ‘defined’ predicted orthogroups (‘defined’ test groups), a gene that was present in both the test group and its corresponding gold group was considered as true positive (TP), whereas a gene that was only present in the test group, but not in the corresponding gold group, was considered as false positive (FP). In general:

(All genes used in the comparison) = FP + TP + FN + TN (1)

We distinguished FP genes into those that are found in the set of corresponding gold groups (FP_in_) and those that are not found in the set of corresponding gold groups (FP_out_):

FP = FP_in_ + FP_out_ (2)

In addition, we distinguished FN genes to those genes belonging to gold groups that are absent from their corresponding test groups (FN_m_) and to those genes belonging to gold groups that were not matched by any test group (FN_nm_). Thus:

FN = FN_m_ + FN_nm_ (3)

We calculated TP, FP_in_, FP_out_, and FN_m_ values by comparing test groups with their corresponding gold groups. Furthermore, in cases where an algorithm predicted fewer test groups than expected based on the number of gold groups (2,723 for all classes, 210 for Class 0, 149 for Class I, 188 for Class II, 219 for Class III, 1,957 for Class IV), we estimated the FN_nm_ value using the equation:

FN_nm_ = (‘number of gold groups’ – ‘number of defined groups’) x ‘average number of genes per gold group’ (4)

We then used the TP, FP and FN values for the ‘defined’ test genes to estimate true positive (TP*), false positive (FP*), and false negative (FN*) values for the ‘undefined’ test genes according to:

TP* = TP x (number of ‘undefined’ test genes / number of ‘defined’ test genes) (5)

FP* = FP x (number of ‘undefined’ test genes / number of ‘defined’ test genes) (6)

FN* = FN x (number of ‘undefined’ test genes / number of ‘defined’ test genes) (7)

Finally, we estimated the numbers of total true positive (tTP), total false positive (tFP), total false negative (tFN) and total true negative (tTN) genes according to:

tTP = TP + TP* (8)

tFP = FP + FP* (9)

tFN = FN + FN* (10)

tTN = ‘number of genes in proteome set’ – tTP – tFP – tFN (11)
